# Black women’s preferences regarding use of mHealth for sexual health support in Chicago, a cross-sectional study

**DOI:** 10.1371/journal.pdig.0001084

**Published:** 2025-11-13

**Authors:** Eleanor E. Friedman, Catherine Desmarais, Samantha A. Devlin, Emily Ott, Sadia Haider, Amy K. Johnson

**Affiliations:** 1 Section of Infectious Diseases and Global Health, Department of Medicine, University of Chicago, Chicago, Illinois, United States of America; 2 Department of OB/GYN, Rush University Medical Center, Chicago, Illinois, United States of America; 3 The Potocsnak Family Division of Adolescent and Young Adult Medicine, Ann & Robert H. Lurie Children’s Hospital, Chicago, Illinois, United States of America; 4 Department of Pediatrics, Northwestern Feinberg School of Medicine, Chicago, Illinois United States of America; Iran University of Medical Sciences, IRAN, ISLAMIC REPUBLIC OF

## Abstract

Black women are disproportionally likely to contract sexually transmitted infections (STIs) including HIV compared to women of other races and ethnicities. It is possible that mobile health (referred to as “mHealth”) strategies, including mobile applications, designed for Black women could provide sexual health support and reduce STI/HIV transmission. We sought to explore acceptability of mHealth strategies among Black women and to identify if preferences varied by age or HIV vulnerability. We surveyed 213 Black women aged 14–64 attending a family planning clinic in Chicago. We asked about mHealth use, desired sources of sexual health information, and mHealth application (app) features. Responses were analyzed as dichotomous variables, with age categorized as ≤24 years of age or ≥25 years of age and HIV vulnerability score categorized as low (<2) or high (≥2). HIV vulnerability was determined based on affirmative answers to the following questions: having had condomless sex (either vaginal or anal) in the past three months, having had an abortion in the past 12 months, having received STI treatment in the past three months, and having had ≥ 2 sex partners in the last three months. Odds ratios and 95% confidence intervals (OR 95% CI) were created using logistic regression models. The majority of participants were interested in using technology as part of their sexual health care (84.5%) and were likely to download an mHealth app (74.7%). Many questions about desirability and interest in app features did not differ by age or HIV vulnerability category. Black women ≥25 years had 7.3 times the odds of rating the inclusion of short videos as an important part of the mHealth app (OR 7.3 95% CI (1.7, 32.4)). Within this population, interest in using a sexual health app was high, suggesting an openness to app development for both sexual health as well as specifically for pre-exposure prophylaxis.

## Author summary

Mobile health (“mHealth”) is the use of mobile technology applications (on smartphones, tablet computers, and other devices) to support healthcare and facilitate communication between patients and providers. mHealth applications (“app”) have been developed for some groups vulnerable to sexually transmitted infections (STIs) and HIV including men who have sex with men, but not for Black women. Little research has been done on Black women’s interest in a sexual health focused mHealth application. We surveyed 213 Black adolescent and adult women attending a family planning clinic in Chicago, Illinois. We asked about their sexual health history, if they had previously used an mHealth app, interest in an mHealth app for sexual health, and what features to include in such an app. Most participants had a history of mHealth use (67.6%), were interested in using technology as part of sexual healthcare (84.5%) and were interested in downloading an mHealth app for sexual healthcare (74.7%). Survey results did not differ much when examined by age group or HIV vulnerability. The high interest in using an mHealth app for sexual healthcare in our participants underlines the importance of app development among this key population.

## Introduction

Sexually transmitted infections (STIs) significantly and disproportionately affect Black women in the United States. According to the Centers for Disease Control and Prevention (CDC), chlamydia rates among Black women are 5 times the rates among white women [1]. Black women also have greater rates of gonorrhea (6.9 times) and are infected with syphilis at a rate 4.7 times greater than their white counterparts [1]. Additionally, there were 36,801 new HIV diagnoses in the United States in 2019; of those new diagnoses, 19% (6,999) were among women [2]. Among women, Black women aged 25–34 are disproportionately affected by HIV. Women aged 25–34 had the highest number of new HIV diagnoses (27%, 1,923), and Black women made up over half (54%, 3,812) of new HIV diagnoses in 2019 [2]. This burden is particularly evident in urban environments that have a high prevalence of HIV such as Chicago, where the rate of Black women living with HIV in 2021 was 15.3 times that of white women [3].

These continued disparities in sexual health among Black women demonstrate the need for interventions specifically tailored for this population to promote health equity and provide sexual health services. Pre-exposure prophylaxis (PrEP) is a safe and highly effective biomedical method to prevent HIV infection [4] and has the potential to become a key HIV prevention strategy for women [5,6]. However, according to the CDC, only 10% of women who could benefit from PrEP were prescribed it in 2019 [2]. One potential way to support the sexual health of Black women, as well as to support PrEP awareness, knowledge, uptake, and adherence is using mobile health (hereafter referred to as “mHealth”) interventions. mHealth interventions are those that provide medical services and public health activities via mobile devices, including through texts or applications (apps). mHealth can be used to promote healthy behaviors, provide information, and refer users to health services. mHealth has also been shown to improve health communication and education for traditionally underserved populations [7].

Thus far, the majority of mHealth interventions around PrEP have been text- and app-based tools primarily designed to address PrEP uptake and adherence among men who have sex with men (MSM). The acceptability of these interventions has been high; however, factors such as age, race, and socioeconomic status influence acceptance of PrEP [8–15]. mHealth interventions need to be culturally competent and meet the particular needs of their target population [16–18]; consequently, apps designed for women must take into account their specific lack of PrEP knowledge and sexual health needs [19]. For example, only 35% of adult Black women at a Chicago family planning clinic reported being aware of PrEP [20]. Integrating a mobile app tailored for Black women to educate, initiate, and maintain PrEP use into sexual health care could support clinical care and address these broad needs, particularly at family planning clinics which are key access points for reaching women [21].

To develop an mHealth intervention for PrEP initiation and adherence among Black women, it is crucial to first explore factors that are desired in an app to ensure it is appropriate and sufficiently addresses the needs of a specific population [22]. As such, the objectives of this study were to a) explore the preferences of Black women without HIV who access care at family planning clinics in Chicago regarding an mHealth app for sexual health care, b) identify desired mHealth features to inform the design and to support uptake and implementation of HIV prevention (i.e., PrEP) mHealth interventions, and c) to determine if these preferences differed by age or the HIV vulnerability of respondents.

## Methods

Data were obtained from two separate cross-sectional surveys conducted at family planning clinics on the south side of Chicago among self-identified Black or African American women. The first survey was among adult women aged 18 and older, and the second survey was among young women and adolescents between 13–24 years of age. Both studies required participants to be English speaking, self-identify as Black or African American, and report sexual activity within the previous 6 months. Both surveys assessed knowledge of PrEP, interest in starting PrEP, and use of mobile phone health programs and desired mHealth intervention features. Information on demographic characteristics and sexual health behaviors were also collected. Questions were worded identically in both surveys, allowing for combination of survey data.

Desirability of features of mHealth applications were assessed by asking, “How important would the following features be in a sexual health mobile phone app?”. Areas queried included “Chat or communication feature with medical providers”, “Privacy”, “Games”, “Rewards”, “Fitness tracker (e.g., steps taken, calories burned, etc.)”, “Chat or communication feature with other App users”, “Short videos”, “Reminders”, “Resources (clinic locations)”, and “Appearance (logo, colors, etc.)”. Response choices for these questions were “Very important”, “Somewhat important”, or “Not important”.

Additionally, we asked about participants’ interest in particular mHealth features, such as reminders, chat features, and educational components. Questions included “How interested are you in using technology as part of your sexual health care?”, “How interested are you in receiving sexual health reminders via text message? For example, receiving reminders about getting screened for STIs, HIV, or to refill sexual health prescriptions like birth control or PrEP.”, “How interested are you in receiving sexual health reminders via email? For example, receiving reminders about getting screened for STIs, HIV, or to refill sexual health prescriptions like birth control or PrEP.”, “How interested are you in receiving sexual health counseling by online chat? By sexual health counseling, we mean being able to ask an expert about questions related to sexual health, for example questions about birth control, condoms, or PrEP.”, “How interested are you in receiving sexual health education via a mobile phone app?”, and “How interested are you in receiving sexual health education via Internet games?”. These questions had three response choices of “Not at all interested”, “Somewhat interested”, or “Very interested”.

Lastly, two questions were asked about past and future mobile health app downloads: “Have you ever downloaded a health-related mobile phone app?” with choices of “Yes” or “No”, and “How likely are you to download a sexual health mobile phone app?”, with choices of “Not at all likely”, “Somewhat likely”, or “Very likely”.

Written informed consent was obtained from all participants, and a $20 gift card was provided to each participant who completed either study. Survey data were collected from 2/1/2018–6/30/2019 using REDCap electronic data capture tools. The Institutional Review Boards from the University of Chicago (IRB17–0984) and Ann & Robert H. Lurie Children’s Hospital of Chicago reviewed and approved these studies.

### Analysis

To understand interest in a sexual health app in this population, descriptive statistics were used to characterize the sample including the median and interquartile range (IQR) for continuous measures and proportions by category for categorical measures. To investigate if preferences for mHealth features differed by age, we examined the effect of age categorized as ≤24 years of age or ≥25 years of age. We also examined if preferences for mHealth features differed by high or low HIV vulnerability scores. To measure this, we created a summary score of positive responses to several questions on sexual behavior. Variables included in the HIV vulnerability score were having had condomless sex (either vaginal or anal) in the past three months, having had an abortion in the past 12 months, having received STI treatment in the past three months, and having had ≥ 2 sex partners in the last three months. HIV vulnerability scores ranged from 0 to 5, with higher scores indicating greater vulnerability. For logistic regression, we used two categories of HIV vulnerability: low (<2) and high (≥2). Logistic regression models were used to produce odds ratios and 95% confidence intervals (OR 95% CI) quantifying the association of age and HIV vulnerability with mHealth app features. All mHealth features were dichotomized into “Interested” versus “Not interested” or “Important” versus “Not important” for odds ratios. Quantitative analysis was conducted in SAS 9.4 (SAS Institute Inc., Cary, NC, USA).

## Results

In total, 213 Black women responded to the surveys. The median age of participants was 24 years, with an interquartile range of 21–28. Nearly all women in the sample had engaged recently in vaginal sex (93.4%), and 10.3% of women had engaged in anal sex in the last three months. Nearly two thirds of women had been tested for an STI in the last three months, and 23.0% of women had undergone an abortion in the last year. Rates of inconsistent condom use were high for both vaginal sex (83.5%) and anal sex (95.5%). When an HIV vulnerability score was calculated, most women in our sample showed low HIV vulnerability with scores of either 0 (14.1%) or 1 (40.4%) (Table 1). HIV vulnerability differed by age, with women who were ≥25 years of age having 0.4 times the odds of a high HIV vulnerability score compared to women aged 24 years or younger (OR 0.4 95% CI (0.2, 0.7)).

**Table 1:** Characteristics of Black women aged 14-64 who attended family planning clinics and were surveyed regarding the use of sexual health mobile phone applications.

Total sample (n = 213)	Number (percentage)
Age categories	
≤ 18	13 (6.1%)
19-24	111 (52.1%)
25-30	45 (21.1%)
≥ 30	40 (18.8%)
Missing	4 (1.9%)
Where do you live	
Northside	11 (5.6%)
Southside	156 (73.2%)
Westside	10 (4.7%)
Outside the city of Chicago	32 (15.0%)
Missing	4 (1.9%)
Had vaginal sex in last three months	
Yes	200 (93.9%)
No	13 (6.1%)
Condom use vaginal sex (branching)	
Always	33 (16.5%)
Not always	167 (83.5%)
Not asked	13
Had anal sex in last three months	
Yes	22 (10.3%)
No	191 (89.7%)
Condom use anal sex (branching)	
Always	1 (4.5%)
Not always	21 (95.5%)
Not asked	191
Any STI treatment in last three months	
Yes	27 (12.7%)
No	184 (86.4%)
Missing	2 (0.9%)
Any STI testing in last three months	
Yes	140 (65.7%)
No	70 (32.9%)
Missing	3 (1.4%)
Number of partners in last three months	
0	6 (2.8%)
1	173 (81.2%)
2	25 (11.7%)
3	7 (3.3%)
≥ 4	1 (0.5%)
Missing	1 (0.5%)
Had an abortion in the last 12 months	
Yes	50 (23.5%)
No	63 (76.1%)
Missing	1 (0.5%)
HIV vulnerability score	
0	30 (14.1%)
1	86 (40.4%)
2	62 (29.1%)
3	24 (11.3%)
4	2 (0.9%)
5	1 (0.5%)
Missing	8 (3.8%)
Heard of PrEP before today	
Yes	93 (43.7%)
No	119 (55.9%)
Missing	1 (0.5%)
How likely are you to start taking PrEP	
Likely	85 (39.9%)
Unlikely or unsure	125 (58.7%)
Missing	3 (1.4%)

* HIV vulnerability score included having condomless sex (either vaginal or anal) in the past three months, having an abortion in the past 12 months, having received STI treatment in the past three months, and having ≥2 sex partners in the last three months.

Most participants (67.6%) had previously downloaded a health-related mobile phone application, and support for using technology as a component of sexual health care was high (84.5%). When asked directly if participants would be interested in downloading a mobile phone app for sexual health, most respondents were interested (74.7%). When the desirability of app features was examined by age group, no significant differences were seen. Sexual health reminders delivered by email showed a trend towards significance for those ≥25 years of age. No significant differences were seen when comparisons were made between women with high HIV vulnerability scores and women with low HIV vulnerability scores (Table 2).

**Table 2:** Responses to questions about desirability of technology and mHealth tools as part of sexual health care by age group and HIV vulnerability among Black women.

Technology questions	Number (percentage)	Age ≥ 25 Odds Ratio (95% CI)	HIV vulnerability score ≥2 Odds Ratio (95% CI)
How interested are you in using technology as part of your sexual health care?			
Not at all interested	31 (14.6%)	Referent	Referent
Interested	180 (84.5%)	1.1 (0.5, 2.4)	1.3 (0.6, 2.8)
Missing	2 (0.9%)	5 (2.4%)	9 (4.2%)
How interested are you in receiving sexual health reminders via text message? *			
Not at all interested	36 (16.9%)	Referent	Referent
Interested	175 (82.2%)	0.7 (0.4, 1.5)	1.7 (0.8, 3.6)
Missing	2 (0.9%)	5 (2.4%)	9 (4.2%)
How interested are you in receiving sexual health reminders via email? *			
Not at all interested	40 (18.8%)	Referent	Referent
Interested	169 (79.3%)	2.1 (0.99, 4.4)	0.95 (0.5, 1.9)
Missing	4 (1.9%)	6 (2.8%)	11 (5.2%)
How interested are you in receiving sexual health counseling by online chat? By sexual health counseling we mean being able to ask an expert a question related to sexual health, for example questions about birth control, condoms, or PrEP.			
Not at all interested	83 (39.0%)	Referent	Referent
Interested	128 (60.1%)	0.96 (0.5, 1.7)	0.95 (0.5, 1.7)
Missing	2 (0.9%)	6 (2.8%)	9 (4.2%)
How interested are you in receiving sexual health education via a mobile phone app?			
Not at all interested	43 (20.2%)	Referent	Referent
Interested	167 (78.4%)	1.4 (0.8, 2.5)	0.7 (0.3, 1.8)
Missing	3 (1.4%)	6 (2.8%)	10 (4.7%)
How interested are you in receiving sexual health education via Internet games?			
Not at all interested	112 (52.6%)	Referent	Referent
Interested	98 (46.0%)	1.4 (0.8, 2.5)	0.8 (0.5, 1.5)
Missing	3 (1.4%)	6 (2.8%)	10 (4.7%)
How likely are you to download a sexual health mobile phone app?			
Not at all likely	51 (23.9%)	Referent	Referent
Likely	159 (74.7%)	0.7 (0.4, 1.3)	0.9 (0.5, 1.7)
Missing	3 (1.4%)	6 (2.8%)	10 (4.7%)
Have you ever downloaded a health-related mobile phone app?			
No	65 (30.5%)	Referent	Referent
Yes	144 (67.6%)	0.9 (0.5, 1.7)	1.2 (0.6, 2.1)
Missing	4 (1.9%)	7 (3.3%)	11 (5.2%)

* For example, receiving reminders about getting screened for STIs, HIV, or to refill sexual health prescriptions like birth control or PrEP.

** Significance was determined using the 95% confidence intervals.

Popular features of sexual health mobile phone applications included privacy (97.2% important), clinic locations (97.2% important), ability to communicate with providers (95.8% important), and reminders (94.8% important). Least popular features included the ability to receive sexual health education via internet games (46.0% important), having games as part of a sexual health app (45.5% important), having the ability to chat or communicate with other users (64.3% important), and rewards (69.0% important). When desirability of specific app features were asked about, women ≥25 years had 7.3 times the odds of rating the inclusion of short videos as part of the mHealth app as important (OR 7.3 95% CI (1.7, 32.4)). Additionally, the inclusion of games showed a trend towards significance for those ≥25 years of age. No differences in features were seen when examining women with low HIV vulnerability scores versus women with high scores (Table 3).

**Table 3:** Questions regarding the importance of various technology features as components of a sexual health app by age group and HIV vulnerability among Black women.

How important would the following features be in a sexual health mobile phone app?	Number (percentage)	Age ≥ 25 Odds Ratio (95% CI)	HIV vulnerability score ≥2 Odds Ratio (95% CI)
Chat or communication feature with medical providers			
Important	204 (95.8%)	0.3 (0.1, 1.8)	0.8 (0.2, 3.9)
Not important	6 (2.8%)	Referent	Referent
Missing	6 (2.8%)	6 (2.8%)	10 (4.7%)
Privacy			
Important	207 (97.2%)	0.7 (0.1, 10.9)	N/A
Not important	2 (0.9%)	Referent	
Missing	4 (1.9%)	7 (3.3%)	
Games			
Important	97 (45.5%)	1.6 (0.9, 2.8)	0.6 (0.3, 1.01)
Not important	113 (53.1%)	Referent	Referent
Missing	3 (1.4%)	6 (2.8%)	10 (4.7%)
Rewards			
Important	147 (69.0%)	1.2 (0.6, 2.1)	0.7 (0.4, 1.4)
Not important	61 (28.6%)	Referent	Referent
Missing	5 (2.4%)	8 (3.8%)	12 (5.6%)
Fitness tracker (e.g., steps taken, calories burned, etc.)			
Important	186 (87.3)	0.6 (0.3, 1.5)	0.7 (0.3, 1.6)
Not important	24 (11.3%)	Referent	Referent
Missing	3 (1.4%)	6 (2.8%)	10 (4.7%)
Chat or communication feature with other app users			
Important	137 (64.3%)	1.2 (0.7, 2.2)	1.6 (0.9, 3.0)
Not important	73 (34.3%)	Referent	Referent
Missing	3 (1.4%)	6 (2.8%)	10 (4.7%)
Short videos			
Important	189 (88.7%)	7.3 (1.7, 32.4)**	0.7 (0.3, 1.7)
Not important	21 (9.9%)	Referent	Referent
Missing	3 (1.4%)	6 (2.8%)	10 (4.7%)
Reminders			
Important	202 (94.8%)	1.1 (0.3, 4.8)	0.5 (0.1, 2.0)
Not important	8 (3.8%)	Referent	Referent
Missing	3 (1.4%)	6 (2.8%)	10 (4.7%)
Resources (clinic locations)			
Important	207 (97.2%)	0.3 (0.03, 3.7)	0.4 (0.03, 4.3)
Not important	3 (1.4%)	Referent	Referent
Missing	3 (1.4%)	6 (2.8%)	10 (4.7%)
Appearance (logo, colors, etc.)			
Important	155 (72.8%)	1.2 (0.7, 2.3)	0.7 (0.4, 1.3)
Not important	55 (25.8%)	Referent	Referent
Missing	3 (1.4%)	6 (2.8%)	10 (4.7%)

** Significance was determined using the 95% confidence intervals.

## Discussion

As previous research regarding mHealth for PrEP support and adherence has focused primarily on MSM, transgender women, and adolescent or young women, this study adds to the literature with its focus on urban Black women of child-bearing age accessing sexual health care at family planning clinics [23–26]. Within this population, responses to the concept of a sexual health app were positive, suggesting an openness to app development for both sexual health as well as specifically for PrEP. Women placed particular value on app appearance, privacy, reminder features, clinic locators, and provider communication tools.

Importantly, when testing if app features differed by age group or HIV vulnerability, few significant differences were seen. Our results suggest that it may be possible to design a single app that would be acceptable to a broad range of Black women regardless of age and/or sexual behaviors. Future work will need to replicate and extend these findings, but if confirmed, this may greatly simplify considerations surrounding application design and tailoring.

Our findings regarding app features are consistent with several other surveys and focus groups intended to elucidate user preferences for sexual health and PrEP apps. Women in this population showed enthusiasm for a provider chat feature in a sexual health app. Chats have a quick response time compared to other communication tools such as email. This finding aligns with similar findings on the popularity of electronic patient portals among patients to contact their medical provider and to examine information from their electronic medical records [27].

Respondents were also interested in apps containing a reminder feature. Daily reminders via text have been found to improve medication adherence in adolescents across a range of diseases [28–31]. In a study of using text or email check-ins to support PrEP adherence in MSM in San Francisco and Chicago, users under age 30 and Black individuals were more likely to use the tool. Furthermore, MSM in Chicago strongly believed the intervention should be available to all persons starting PrEP [11]. An expansion of this study in Chicago found that MSM and transgender women initiating PrEP within Chicago’s safety-net healthcare system who were assigned to the intervention were more likely to attend their follow-up visits and adhere to PrEP [10]. Of note, these reminder programs were overwhelmingly text- instead of app-based, and PrEP-specific interventions were focused on MSM and transgender women. Therefore, research is needed on app-based reminder systems and systems targeted at women to determine notification preferences and impact on adherence.

Privacy is important to sexual health app users. Participants in other studies, as well as our own, have expressed concern about others seeing texts, app names, emails, or notifications on their phones [32,33]. To address this barrier, password protection with a timeout feature could be incorporated. The app design, including the name of the app, should be discrete and not refer to sexual health. Furthermore, the app should have the ability to be hidden, utilizing a “secret app” function or false icon [32,34]. Participants have also expressed a preference for giving minimal personal information when signing up for a sexual health app, citing concerns about who will have access to potentially sensitive and identifying information [32]. Consistent with prior studies, the ability to look up the locations of nearby clinics was desired by respondents. This feature can be tailored to find specific services such as STI treatment, contraception, abortion, and social services [35]. There are currently service locator programs being maintained, such as the PrEP Locator [36], thus requiring less maintenance of this feature in future apps developed to support sexual health and PrEP use.

Our participants deemed some app features unimportant (e.g., gamification, rewards, and the ability to interact with peers). Previous studies on sexual health apps with gamification and real-world rewards have shown these are features of interest, especially among adolescents. One other study conducted among Black women found similar results [24]; however, more work among Black women will need to be conducted to determine the consistency of our findings related to gamification and rewards.

This study has several limitations, including a relatively small sample size among Black women, with most participants being aged 19–24. Our findings are less likely to be generalizable as we used a convenience sample of participants at different family planning clinic locations. While these clinics are in the same geographic area, there may be differences between them that affected our results. Finally, caution should be taken if extrapolating these results as the opinions expressed in this study may not represent the full spectrum of preferences among urban Black women.

## Conclusion

We examined interest in and desired features of a sexual health app for Black women who attended a family planning clinic in Chicago. Responses to the concept of a sexual health app were positive, indicating an openness to app development for both sexual health and for PrEP specifically. This study identified the preferences and needs for an mHealth application tailored to Black women in Chicago, a population that has increased vulnerability to STIs and HIV. Future studies should seek to replicate the preferences that we identified, as well as expanding to other geographic areas to better represent urban Black women. Additional work to ensure a complete understanding of preferences, barriers to accessing healthcare, and other social determinants of health will be needed to ensure a culturally appropriate and user-friendly app for Black women in the United States.
